# Efficient visible-light-driven photocatalytic detoxification of a sulfur mustard simulant in air using rose bengal-functionalized MOFs†

**DOI:** 10.1039/d5ra02657a

**Published:** 2025-07-14

**Authors:** Jinfeng Zhou, Xiaodong Zhang, Kesheng Cao, Qing Zhou, Jinping Cao, Renpeng Guan, Chunjie Chu

**Affiliations:** a College of Chemistry and Environmental Engineering, Pingdingshan University Pingdingshan 467000 P. R. China zhoujf016@163.com; b Yaoshan Laboratory Pingdingshan 467000 P. R. China; c Henan Province Engineering Technology Research Center of Green Hydrogen & Electrochemical Energy Storage P. R. China; d Fujian Engineering Research Center of Advanced Manufacturing Technology for Fine Chemicals, College of Chemical Engineering, Fuzhou University Fuzhou 350116 P. R. China

## Abstract

Sulfur mustard, a highly toxic chemical warfare agent, poses a significant threat to human health. Consequently, the development of efficient and rapid decontamination strategies is of paramount importance. However, current degradation methods are often hindered by slow reaction rates and limited selectivity. Herein, we report a facile one-pot *in situ* self-assembly method to simultaneously modify the photosensitizer rose bengal (RB) into both the cavities and surface of zeolitic imidazolate framework-8 (ZIF-8), resulting in the formation of the RB@ZIF-8 composite. The RB@ZIF-8 composite demonstrates exceptional singlet oxygen (^1^O_2_) photosensitization capacity, serving as a visible-light-driven heterogeneous photocatalyst that enables selective oxidation of a sulfur mustard simulant (2-chloroethyl ethyl sulfide, CEES) to the corresponding non-toxicity sulfoxide derivative. This system achieves complete conversion within 6 minutes, with a reaction half-life of 2.5 minutes under ambient conditions. Moreover, the composite demonstrates outstanding recyclability and reusability. This work provides a promising strategy for the design of advanced MOF-based heterogeneous photosensitizers, offering a highly efficient, selective, and reusable platform for the rapid detoxification of sulfur mustard under mild conditions.

## Abstract 2

A novel visible-light-driven heterogeneous photosensitizer RB@ZIF-8 composite was successfully synthesized, which can detoxify the sulfur mustard simulant (CEES) into non-toxic sulfoxide within 6 minutes under mild conditions.

## Graphical abstract

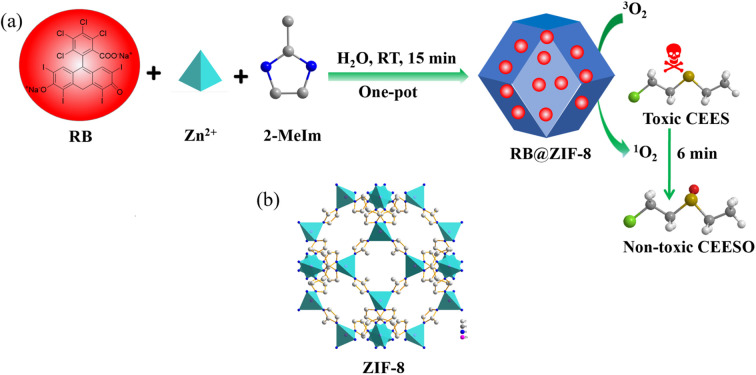

## Introduction

Mustard gas [bis(2-chloroethyl) sulfide, commonly referred to as HD] is an extremely toxic vesicant chemical warfare agent.^1,2^ Exposure to HD can cause severe skin blistering, respiratory tract irritation, eye damage, and may be lethal at high doses.^3–5^ Therefore, it is critically important to develop strategies for the rapid and efficient detoxification of HD into non-toxic products. Recent studies have identified three major detoxification pathways for sulfur mustard (HD): oxidation,^6–10^ hydrolysis,^11–14^ and dehydrohalogenation.^15,16^ However, the slow reaction rates of hydrolysis and dehydrohalogenation render them unsuitable for practical applications.^17^ Selective oxidation has consequently emerged as a promising strategy for the detoxification of sulfur mustard. This method efficiently converts HD into less toxic sulfoxides, while avoiding excessive oxidation that could lead to the formation of highly toxic sulfones.^18,19^ Singlet oxygen (^1^O_2_), a mild and highly selective reactive oxygen species, has demonstrated exceptional efficiency in selectively oxidizing HD to sulfoxides without producing highly toxic sulfones.^6–10,17–19^ As a result, it has garnered significant attention as a promising strategy in the field of HD detoxification.

Rose bengal (RB), a commercially available photosensitizer, has garnered significant attention in photodynamic therapy and photocatalysis due to its remarkable efficiency in generating ^1^O_2_.^20–22^ However, the conjugated π–π electronic structure of RB molecules makes them prone to self-aggregation, thereby reducing light absorption efficiency and significantly inhibiting ^1^O_2_ generation.^23^ Furthermore, the challenge of recovering free RB molecules after reactions significantly restricts their practical applications. Therefore, immobilizing RB onto a solid support to develop heterogeneous photosensitizers has emerged as an effective strategy to address these challenges. Among various candidates, metal–organic frameworks (MOFs), characterized by their high specific surface area, permanent porosity, and highly tunable structures, are widely recognized as ideal carriers for the development of heterogeneous photosensitizers.^24–27^ The high surface area and adjustable pore sizes of MOFs facilitate the uniform dispersion of RB molecules, while their porous structures significantly enhance substrate and product diffusion during catalytic reactions. Thus, the integration of RB with MOFs not only overcomes the limitations of RB in homogeneous systems but also paves the way for the development of efficient and reusable heterogeneous photosensitizers with enhanced catalytic performance.

Zeolitic imidazolate framework-8 (ZIF-8), a prototypical metal–organic framework (MOF) material with a zeolite-like structure, possesses a porous architecture consisting of 11.6 Å cavities and 3.4 Å apertures.^28^ Owing to its exceptional chemical and thermal stability, as well as its ease of synthesis, ZIF-8 has been extensively studied as a versatile catalyst support in a wide range of applications.^29–34^

In this study, the well-known photosensitizer rose bengal (RB) was selected as the guest molecule, and ZIF-8 was chosen as the host material to design and fabricate a novel RB@ZIF-8 composite. Using a simple one-pot *in situ* self-assembly method, RB molecules were encapsulated within the cavities of ZIF-8 and adsorbed onto its surface in an aqueous solution under ambient conditions. The RB@ZIF-8 composite was thoroughly characterized using powder X-ray diffraction (PXRD), Fourier-transform infrared spectroscopy (FT-IR), transmission electron microscopy (TEM), UV-Vis spectroscopy, X-ray photoelectron spectroscopy (XPS), and Brunauer–Emmett–Teller (BET) analysis. The ^1^O_2_ generation capability of the composite was systematically evaluated, revealing that the RB@ZIF-8 composite effectively retains the efficient visible-light-driven ^1^O_2_ production of RB. As a result, the RB@ZIF-8 composite emerges as a high-performance, visible-light-driven heterogeneous photosen-sitizer with significant potential for the efficient photocatalytic detoxification of sulfur mustard simulants in air.

## Experimental

### Materials and instruments

Unless otherwise specified, all reagents were obtained from commercial sources and used without further purification. UV-Vis absorption spectra in solution were recorded using a UV-2550 spectrophotometer, while UV-Vis diffuse reflectance spectra in solid were measured on the same instrument equipped with an integrating sphere using BaSO_4_ as a reference standard. Fourier transform infrared (FT-IR) spectra were acquired on a Nicolet 380 FT-IR spectrometer using KBr discs over a range of 4000–400 cm^−1^. Powder X-ray diffraction (PXRD) patterns were recorded on a Bruker D8 Advance diffractometer using Cu Kα radiation (*λ* = 1.5406 Å). Nitrogen adsorption measurements were performed using a Micromeritics 3Flex automated micropore analyser. Transmission electron microscopy (TEM) images were obtained using an FEI Tecnai G2 F20 microscope. Electron paramagnetic resonance (EPR) spectra were acquired using a Bruker EMX nano spectrometer. The particle size and zeta potential were measured using a Malvern Zetasizer Nano equipped with a dynamic light scattering analyzer. X-ray photoelectron spectroscopy (XPS) was performed using a Thermo Scientific ESCALAB 250 Xi spectrometer with monochromatic Al Kα radiation. Proton nuclear magnetic resonance (^1^H NMR) spectra were recorded on a Bruker AM 400 MHz spectrometer.

### Synthesis of RB@ZIF-8 composite

RB@ZIF-8 composite was synthesized *via* a one-pot method according to our published literature.^6^ Initially, RB (10.0 mg, 0.010 mmol) was dissolved in 1.0 mL of H_2_O, while Zn(NO_3_)·6H_2_O (0.20 g, 0.67 mmol) was dissolved in 1.0 mL of H_2_O, and 2-methylimidazole (2.0 g, 4.62 mmol) was dissolved in 8.0 mL of H_2_O. The RB and Zn(NO_3_)·6H_2_O solutions were then combined under continuous stirring. Subsequently, the 2-methylimidazole solution was added dropwise to the mixture at room temperature. After 15 minutes, the resulting rose-red product was collected by centrifugation, followed by sequential wash with water and ethanol until the supernatant became colorless and the absorption spectrum of RB was no longer undetectable. The final powder product was vacuum-dried at room temperature.

For comparison, ZIF-8 was synthesized following a procedure similar to that used for RB@ZIF-8, except without the addition of RB molecules.

### The ^1^O_2_ generation capability of RB@ZIF-8 composite

DPBF exhibits a characteristic absorption peak at 410 nm. Its reaction with ^1^O_2_ produces a colorless product, causing a decrease in the DPBF absorption spectrum. Consequently, the ability of RB@ZIF-8 to generate ^1^O_2_ can be assessed by monitoring the changes in the DPBF absorption spectrum. The procedure is as follows: 6 mg of RB@ZIF-8 composite was dispersed in 5 mL of methanol, followed by the addition of 10 μL of DPBF solution (10 mmol L^−1^). The mixture was subjected to ultrasonication to ensure uniform dispersion. The resulting suspension was then irradiated with a green LED light for 5 seconds, followed by centrifugation to separate the supernatant. The absorption spectrum of the supernatant was recorded, after which the testing solution was returned to the reaction system for ultrasonic treatment, irradiation, and centrifugation. The supernatant was subsequently collected, and its absorption spectrum was measured again. This process was repeated several times.

### General procedure for photocatalytic oxidation of DHN

DHN can be oxidized by ^1^O_2_ to form 5-hydroxy-1,4-naphthoquinone (juglone), a reaction characterized by a decrease in the absorption spectrum of DHN and a corresponding increase in that of juglone. Thus, the generation of ^1^O_2_ can be evaluated by tracking the spectral changes of DHN. The experimental procedure was carried out as follows: 6 mg of RB@ZIF-8 composite was ultrasonically dispersed in 10 mL of a 2 × 10^−4^ mol L^−1^ DHN ethanol solution to prepare a uniform suspension. This suspension was irradiated with a green LED for 20 minutes, after which it was centrifuged to separate the supernatant. The absorption spectrum of the supernatant was subsequently measured. Following each measurement, the supernatant was reintroduced into the catalytic system, and the irradiation and centrifugation steps were repeated.

### General procedure for selective photooxidation of CEES

20 mg of RB@ZIF-8 composite was ultrasonically dispersed in 1.5 mL of methanol to obtain a uniform suspension, which was then transferred to a 10 mL reaction vial. Subsequently, 0.5 mmol of CEES (20 μL) was directly added to the suspension. The mixture was then irradiated with a green LED light (50 mW cm^−2^) for different time. After the reaction, the solvent was removed under reduced pressure, and the residue was collected directly for characterization using ^1^H NMR spectroscopy without further purification. The peak intensity ratio of the substrate and product in the ^1^H NMR spectrum was used to calculate the conversion rate.

## Results and discussion

### Synthesis and structural characterization of RB@ZIF-8 composite

**Fig. 1:**
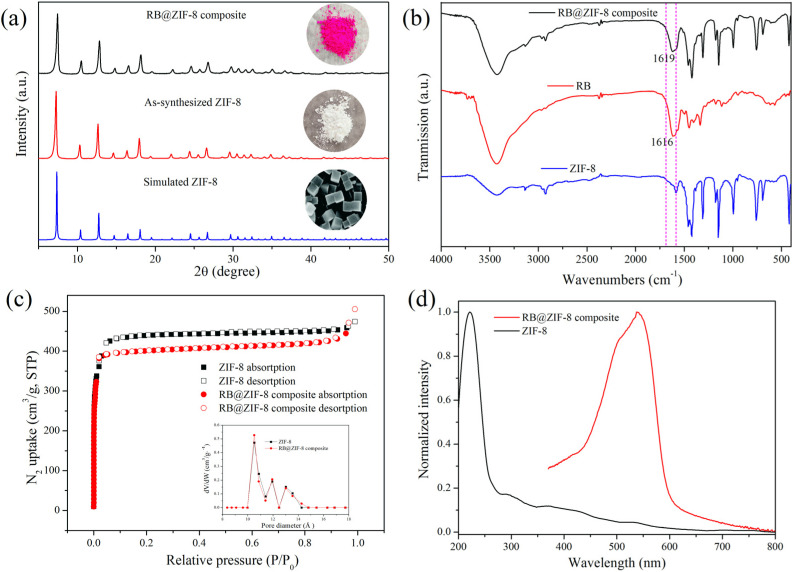
(a) PXRD patterns of RB@ZIF-8 composite, as-synthesized ZIF-8 and simulated ZIF-8. The photographs of RB@ZIF-8 composite, as-synthesized ZIF-8 and simulated ZIF-8 are shown in the inset. (b) FT-IR spectra of RB@ZIF-8 composite, RB and as-synthesized ZIF-8. (c) N_2_ sorption isotherms of RB@ZIF-8 composite and ZIF-8 at 77 K and their pore size distribution (Inset). (d) UV-Vis diffused reflectance spectra of RB@ZIF-8 composite and ZIF-8 powder.

**Scheme 1:**
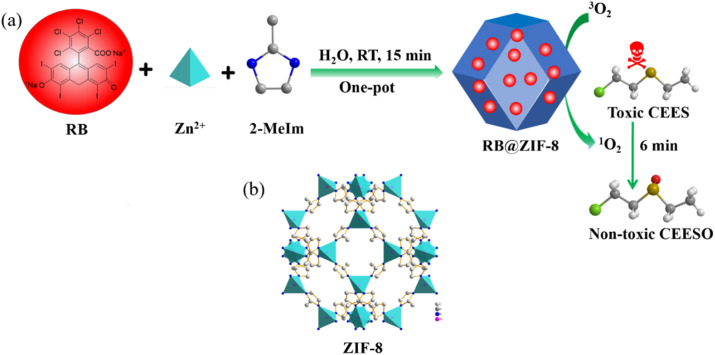
(a) The synthesis process of RB@ZIF-8 composite and its application as a visible light-driven heterogeneous photosensitizer for the efficient detoxification of a sulfur mustard simulant in air. (b) The crystal structure of ZIF-8.

RB is widely recognized for its exceptional ability to generate ^1^O_2_ under visible light irradiation, making it an ideal photosensitizer. Meanwhile, ZIF-8, characterized by its large pore size, excellent chemical stability, and ease of synthesis, serves as an optimal host material. In this study, a simple one-pot *in situ* self-assembly method was utilized to uniformly incorporate RB molecules into the pores and onto the surface of ZIF-8, resulting in the successful synthesis of RB@ZIF-8 composite, as illustrated in Fig. 1. Specifically, RB, Zn(NO_3_)_2_·6H_2_O and 2-MeIm were mixed and stirred in an aqueous solution at room temperature, and the synthesis was completed within just 15 minutes. The resulting precipitate was separated by centrifugation and repeatedly washed with H_2_O and ethanol until the supernatant became colorless. The product was then dried to obtain a rose-red powder, identified as RB@ZIF-8 composite. This one-pot synthesis strategy, compared to solvothermal methods, is simpler, more cost-effective, and environmentally friendly, providing a green and efficient route for the synthesis of MOF-based heterogeneous photosensitizer composites (Scheme 1).

The RB@ZIF-8 composite was comprehensively characterized using PXRD, FT-IR, TEM, BET analysis, UV-Vis absorption spectroscopy, zeta potential and XPS. The PXRD results demonstrated that the characteristic diffraction peaks of the synthesized ZIF-8 and RB@ZIF-8 composite were in excellent agreement with the simulated data for ZIF-8 single crystals (Fig. 1a), confirming the successful synthesis of ZIF-8 and and that the incorporation of RB did not disrupt its framework structure. Furthermore, as shown in the inset of Fig. 1a, the color changed from the white of ZIF-8 to rose-red of RB@ZIF-8 composite under natural light, indicating the successful incorporation of RB guest molecules into ZIF-8. After multiple washes with water and ethanol, the composite retained its color, further demonstrating the strong binding of RB molecules to the ZIF-8 framework. As illustrated in Fig. 1b, FT-IR spectroscopy reveals a characteristic peak of RB at 1616 cm^−1^, corresponding to the stretching vibrations of C=C bonds. In the RB@ZIF-8 composite, this peak shifts slightly to 1619 cm^−1^, indicating the successful loading of RB molecules onto the ZIF-8 framework. Furthermore, the distinct characteristic absorption peaks of ZIF-8 remain unchanged in the composite, confirming that the structural integrity of the ZIF-8 framework is preserved during the modification process. As shown in Fig. 1c, N_2_ adsorption–desorption isotherm studies at 77 K revealed that both ZIF-8 and RB@ZIF-8 composite exhibited typical type I isotherms, confirming their microporous nature. However, the incorporation of RB reduced the specific surface area from 1938 m^2^ g^−1^ for ZIF-8 to 1785 m^2^ g^−1^ for RB@ZIF-8 composite, suggesting that a portion of the pore space was occupied by RB molecules. The solid-state UV-Vis diffuse reflectance spectrum of RB@ZIF-8 composite (Fig. 1c and S2†) displays a distinct RB absorption band at approximately 540 nm in the visible region, confirming the successful incorporation of RB into the ZIF-8 framework. Furthermore, the RB molecules predominantly exist in monomeric form within the composite, as the dimeric form exhibits a blue shift to approximately 514 nm.^21^ The TGA curves demonstrate that the thermal stability of RB@ZIF-8 composite lies between that of pure RB and ZIF-8 (Fig. S3†). The lower weight loss of RB@ZIF-8 composite compared to pure RB suggests that the RB molecules are effectively encapsulated or adsorbed onto the ZIF-8 framework, providing enhanced thermal stability and confirming the successful incorporation of RB into the ZIF-8 structure. Beyond functioning as a support material, ZIF-8 serves as a multifunctional platform for heterogeneous catalysis. Its exceptionally high surface area and tunable porosity facilitate the effective immobilization and dispersion of RB molecules both within the cavities and on the surface, preventing aggregation caused by π–π stacking interactions (Fig. 1d). This structural confinement maintains the monomeric state of RB and maximizes the accessibility of active sites, which is crucial for sustaining photocatalytic efficiency.

To verify whether RB can be modified onto the surface of ZIF-8, a series of experiments were conducted. The zeta potential of pristine ZIF-8 was measured to be +21.2 mV, confirming its positively charged surface. As an anionic dye, RB readily adsorbs onto the ZIF-8 surface through electrostatic interactions. After loading RB, the zeta potential of the RB@ZIF-8 composite significantly decreased to −26.7 mV (Fig. S4†), indicating the successful adsorption of RB onto the ZIF-8 surface and a substantial change in surface charge due to the incorporation of the negatively charged dye. To further validate this conclusion, a mixture of ZIF-8 and RB was prepared. Specifically, 10 mg of RB and 100 mg of ZIF-8 were stirred in 10.0 mL of H_2_O at room temperature (RT) for 5 hours. The mixture was centrifuged and thoroughly washed with water until the supernatant became colorless. The experimental results indicated that the product exhibited a rose-red color, confirming the adsorption of RB molecules onto the surface of ZIF-8 (Fig. S5†). Overall, the findings clearly demonstrate that RB molecules can be encapsulated within the porous structure of ZIF-8 as well as adsorbed onto its external surface.

**Fig. 2:**
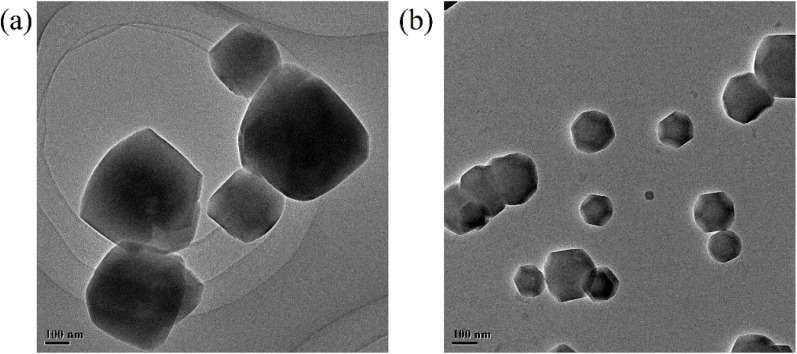
TEM images of ZIF-8 (a) and RB@ZIF-8 composite (b).

In addition to the aforementioned characterization analyses, TEM and SEM images were employed to investigate the morphology of ZIF and the RB@ZIF-8 composite. The results reveal that ZIF and the RB@ZIF-8 composite possess similar morphology and size (Fig. 2, S6 and S7†), indicating that the incorporation of RB molecules does not affect the structural integrity.

**Fig. 3:**
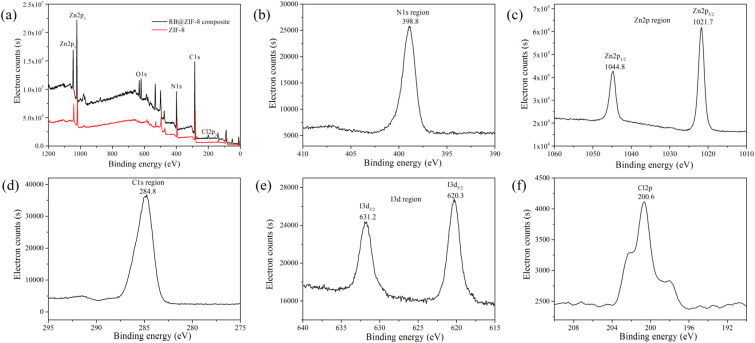
XPS spectra: (a) full range scan of RB@ZIF-8 composite and ZIF-8. (b) N 1s region of RB@ZIF-8 composite. (c) Zn 2p region of RB@ZIF-8 composite; (d) C 1s region of RB@ZIF-8 composite. (e) I 3d region of RB@ZIF-8 composite; (f) Cl 2p region of RB@ZIF-8 composite.

X-ray photoelectron spectroscopy (XPS) was also utilized to characterize the elemental composition of the RB@ZIF-8 composite. The XPS spectra (Fig. 3) display characteristic peaks corresponding to Zn 2p, C 1s, N 1s, and O 1s in both ZIF-8 and RB@ZIF-8 composite, confirming the structural integrity of the ZIF-8 framework. Notably, the appearance of I 3d (Fig. 3e) and Cl 2p (Fig. 3f) peaks from RB molecules in RB@ZIF-8 composite confirms the incorporation of RB molecules. These results clearly demonstrate the modification of ZIF-8 with RB molecules and the formation of the RB@ZIF-8 composite.

### ^1^O_2_ generation of RB@ZIF-8 composite

**Fig. 4:**
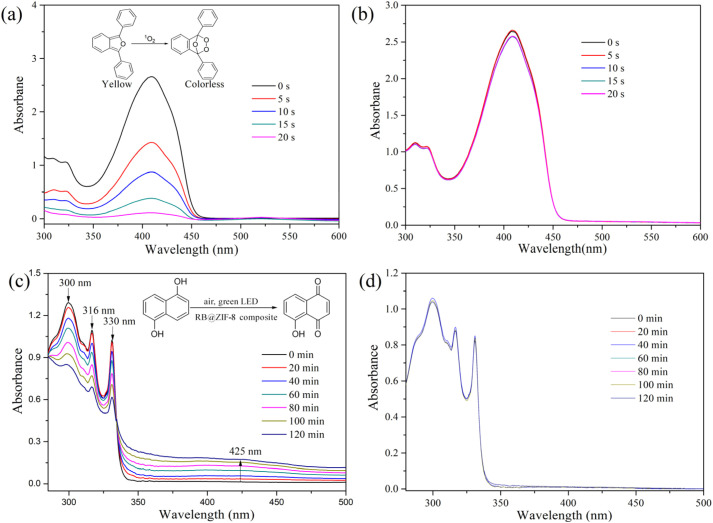
(a) Time-dependent absorption spectra of DPBF upon a green LED irradiation in the presence of RB@ZIF-8 composite. (b) Time-dependent absorption spectra of DPBF upon a green LED irradiation in the presence of ZIF-8 composite. (c) Time-dependent absorption spectra of 1,5-DHN upon a green LED irradiation in the presence of RB@ZIF-8 composite. (d) Time-dependent absorption spectra of 1,5-DHN upon a green LED irradiation in the presence of ZIF-8 composite.

Rose bengal (RB) has gained significant attention for its efficient generation of ^1^O_2_ under visible light irradiation. To assess whether RB@ZIF-8 composite retains this capability, we evaluated its ^1^O_2_ production using DPBF as an indicator. DPBF reacts with ^1^O_2_ to produce a colorless product, leading to a reduction in its absorption spectrum, which enables quantitative analysis of ^1^O_2_ generation. It can be seen from Fig. 4a that a significant decrease in DPBF absorption was observed in the presence of RB@ZIF-8 composite, light and air, whereas minimal changes were detected with ZIF-8 under the same conditions. These results confirm the outstanding capability of RB@ZIF-8 composite to generate ^1^O_2_. To further verify this, DHN was also employed as a probe. DHN is known to undergo oxidation by ^1^O_2_, producing 5-hydroxy-1,4-naphthoquinone (juglone), a transformation that can be tracked *via* changes in its absorption spectrum. The results showed a marked reduction in DHN absorption peaks at 316 nm and 330 nm, accompanied by a significant increase in the juglone absorption peak at 425 nm in the presence of RB@ZIF-8 composite, light, and air (Fig. 4c). In contrast, ZIF-8 exhibited negligible changes under identical conditions (Fig. 4d). These findings strongly confirm the efficient ^1^O_2_ generation by RB@ZIF-8 composite. The ^1^O_2_ generation ability of RB@ZIF-8 composite was also evaluated using electron paramagnetic resonance (EPR) spectroscopy with TEMP as the spin-trapping agent. Upon visible light irradiation of the RB@ZIF-8 composite and TEMP mixture, a distinct 1 : 1 : 1 triplet signal corresponding to the TEMP-^1^O_2_ adduct was observed (Fig. S8†). In contrast, no significant signal was detected in the absence of the composite. These findings confirm the excellent ^1^O_2_ generation capability of RB@ZIF-8 composite, underscoring its superior photosensitizing performance and promising potential for diverse applications.

### Photocatalytic detoxification of a sulfur mustard simulant

**Fig. 5:**
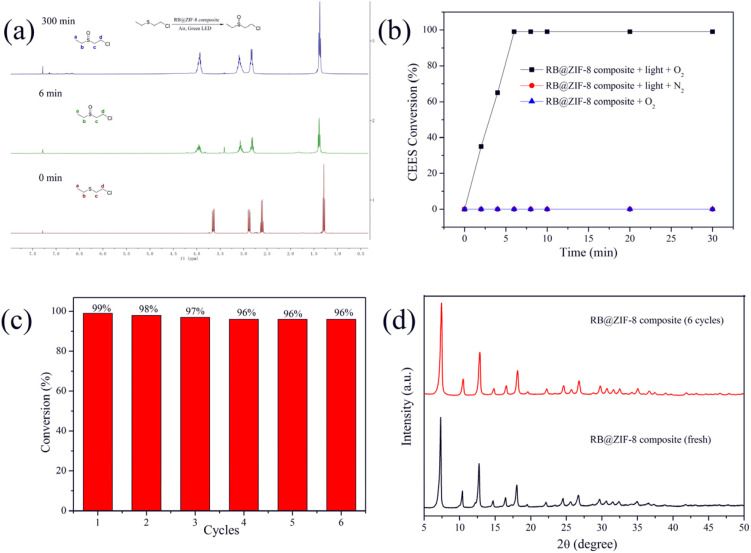
(a) 1H NMR spectrum detection of photooxidation reaction of CEES catalyzed by RB@ZIF-8 composite. (b) Time-dependent conversion of CEES catalyzed by RB@ZIF-8 composite. (c) Recyclability of RB@ZIF-8 composite in the photocatalytic detoxification of CEES. (d) PXRD patterns of the RB@ZIF-8 composite before and after six cycles of CEES degradation.

Research has demonstrated that sulfur mustard (HD) and its simulant, 2-chloroethyl ethyl sulfide (CEES), can be selectively oxidized by ^1^O_2_ to produce less toxic sulfoxide compounds. Due to the high toxicity of HD, CEES is widely employed as a safer alternative in laboratory studies. In this study, the RB@ZIF-8 composite was utilized for the detoxification of CEES, with the reaction process monitored in real time *via*^1^H NMR spectroscopy. As shown in Fig. 5a, under ambient conditions, CEES was completely converted to CEESO within 6 minutes, as evidenced by the disappearance of CEES signals at 42.9, 33.7, 26.1, and 14.6 ppm, and the appearance of CEESO signals at 1.37, 2.80, 3.07, and 3.93 ppm. Notably, even after extending the reaction time to 360 minutes, the highly toxic by-product CEESO_2_ was not detected. Kinetic analysis revealed that the detoxification reaction had a half-life of approximately 2.5 minutes (Fig. 5b), which is significantly faster than most MOF-based photocatalysts reported to date (Table S1†). These findings underscore the exceptional catalytic performance of the RB@ZIF-8 composite in CEES photodegradation.

To assess the reusability of the RB@ZIF-8 composite, multiple catalytic cycles were conducted by continuously adding CEES to the reaction system without separation. As shown in Fig. 5c, the composite exhibited stable catalytic activity over six consecutive CEES detoxification cycles without any significant decrease, demonstrating excellent recyclability. ZIF-8's chemical robustness ensures structural integrity during catalysis. To further evaluate its stability, post-catalysis analyses, PXRD and UV-Vis absorption spectroscopy (Fig. 5d and S8†) were conducted. The results revealed no significant changes in the PXRD patterns or absorption spectra compared to the original sample, suggesting the framework remains intact after multiple cycles, preventing RB leaching. The interconnected pore channels (∼3.4 Å apertures) further facilitate rapid diffusion of CEES and ^1^O_2_, eliminating mass transfer limitations.Therefore, compared to previously reported systems, the RB@ZIF-8 composite exhibits several significant advantages: (1) it achieves complete conversion of CEES to its non-toxic sulfoxide within just 6 minutes, significantly outperforming most MOF-based photocatalysts in terms of reaction rate; (2) it operates efficiently under visible light, which is more practical and environmentally benign than UV irradiation; it exhibits excellent recyclability, maintaining high catalytic activity over multiple reaction cycles with minimal performance loss; and (4) it can be synthesized under mild conditions through a straightforward room-temperature stirring process, offering simplicity and scalability for practical applications.

### Photocatalytic detoxification mechanism of CEES

To mechanistically investigate the involvement of reactive oxygen species (ROS) in CEES detoxification, controlled quenching experiments were conducted using carotenoids as ^1^O_2_ scavengers in the photocatalytic system. The observed marked reduction in CEES-to-CEESO conversion efficiency (from 99% to <1%) upon ^1^O_2_ scavenging provides direct experimental evidence that the detoxification pathway is predominantly mediated by ^1^O_2_ (Fig. S10†). Based on the CEES degradation mechanism reported in the literature, Scheme S1† depicts the proposed reaction pathway for the selective photooxidation of CEES mediated by the RB@ZIF-8 composite. Under visible light irradiation, the photoexcited RB@ZIF-8 composite effectively generates ^1^O_2_, enabling the reaction with CEES molecules to produce a sulfoxide peroxide intermediate. This intermediate undergoes nucleophilic addition with a second CEES molecule, yielding an unstable high-valent anionic species, which rapidly decomposes into the final oxidation product, CEESO.

## Conclusions

In summary, we have successfully developed a visible-light-driven photocatalytic system for the efficient detoxification of a sulfur mustard simulant (CEES) under ambient conditions using a rose bengal-functionalized metal–organic framework (RB@ZIF-8). The RB@ZIF-8 composite can efficiently generate ^1^O_2_ under visible light, enabling the selective conversion of CEES into the less toxic sulfoxide within 6 minutes, while effectively preventing overoxidation to the highly toxic sulfone. Beyond its remarkable catalytic efficiency, the composite exhibits outstanding reusability and structural stability, underscoring its significant potential for the detoxification of chemical warfare agents. This study represents a significant advancement in the design and application of MOF-based photocatalysts, offering a novel and scalable strategy for the efficient defense against chemical warfare agents.

## Conflicts of interest

There are no conflicts to declare.

## Supplementary Material

RA-015-D5RA02657A-s001

## Data Availability

All the data supporting this article are available in the main manuscript or ESI.†
