# Supplementary material for: Efficient visible-light-driven photocatalytic detoxification of a sulfur mustard simulant in air using rose bengal-functionalized MOFs
Source: RSC Adv. 2025 Jul 14;15(30):24557–64. doi: 10.1039/d5ra02657a (PMC12257297; doi:10.1039/d5ra02657a)
Supplement: RA-015-D5RA02657A-s001 [file RA-015-D5RA02657A-s001.pdf]

Supplementary Information (SI) for RSC Advances.
This journal is © The Royal Society of Chemistry 2025

Efficient visible-light-driven photocatalytic detoxification of a sulfur mustard simulant in air using Rose Bengal-functionalized MOFs

Jinfeng Zhou*[a,b,c], Xiaodong Zhang*[a,b], Kesheng Cao[a,b], Qing Zhou[d], Jinping Cao[a], Renpeng Guan[a], Chunjie Chu[a]

[a] College of Chemistry and Environmental Engineering, Pingdingshan University, Pingdingshan 467000, P. R. China

[b] Yaoshan Laboratory, Pingdingshan 467000, P. R. China

[c] Henan Province Engineering Technology Research Center of Green Hydrogen & Electrochemical Energy Storage

[d] Fujian Engineering Research Center of Advanced Manufacturing Technology for Fine Chemicals, College of Chemical Engineering, Fuzhou University, Fuzhou 350116, P. R. China

Corresponding author: zhoujf016@163.com

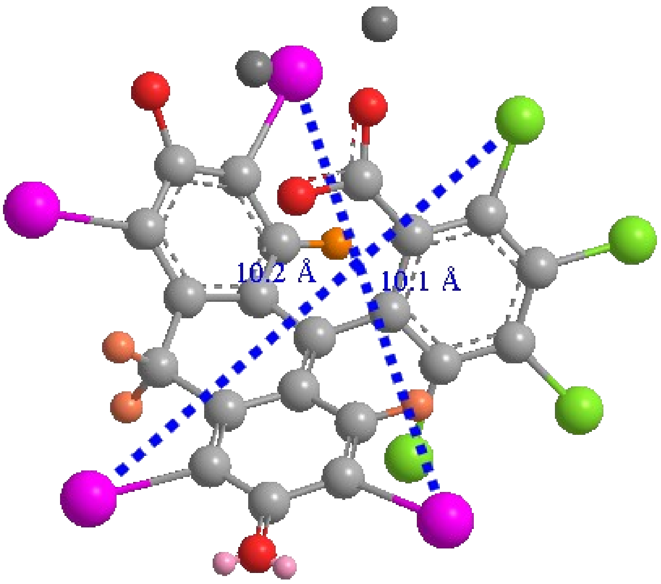

Fig. S1 The structure of RB.

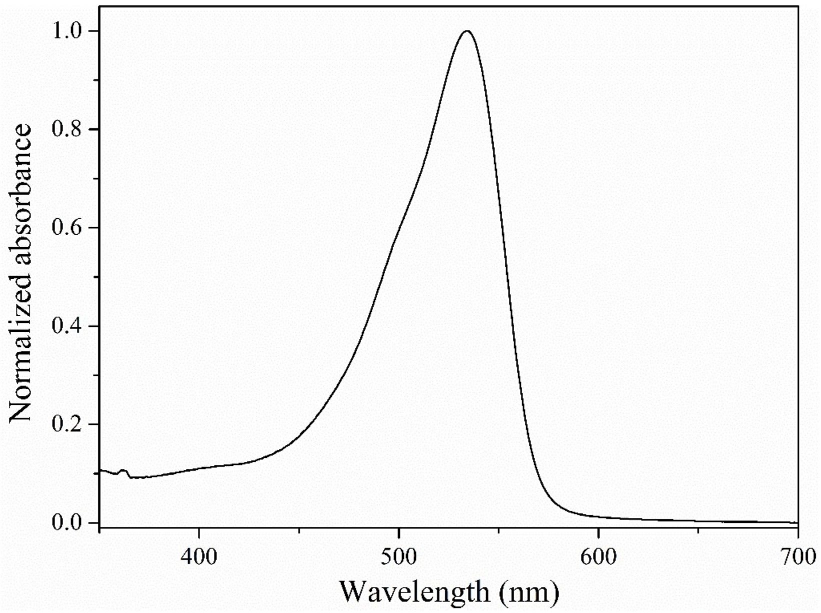

Fig. S2 UV-Vis absorption spectrum of RB solution in H_2O.

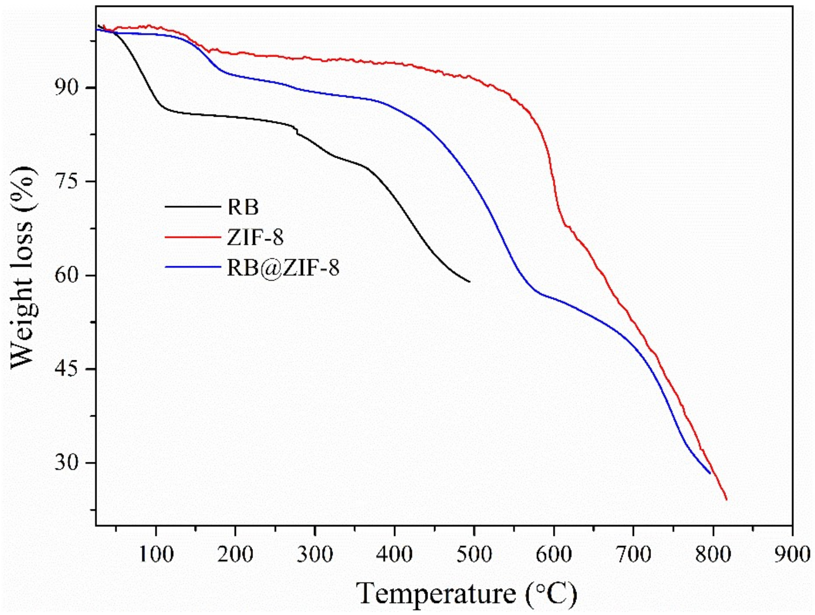

Fig. S3 TGA curves of RB, ZIF-8 and RB@ZIF-8 composite.

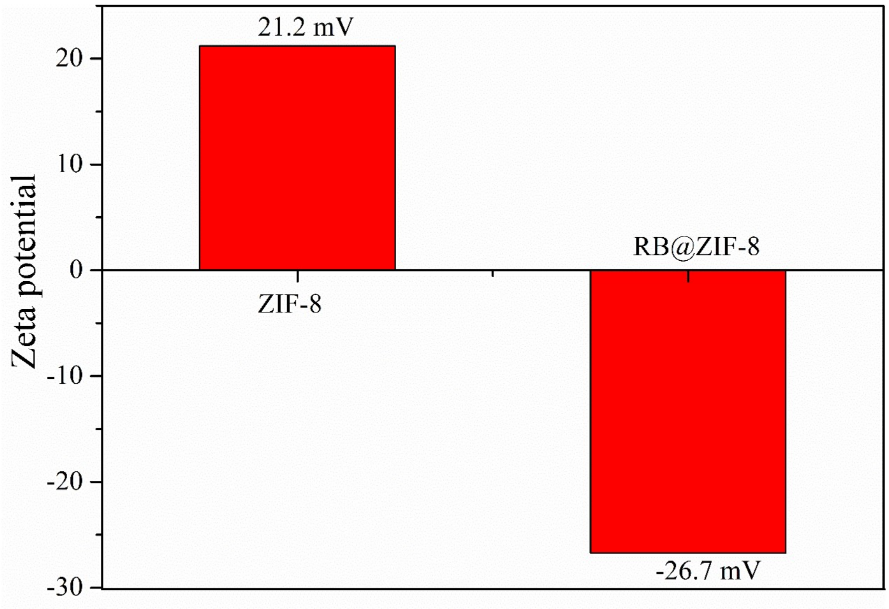

Fig. S4 The zeta potential of ZIF-8 and the RB@ZIF-8 composite.

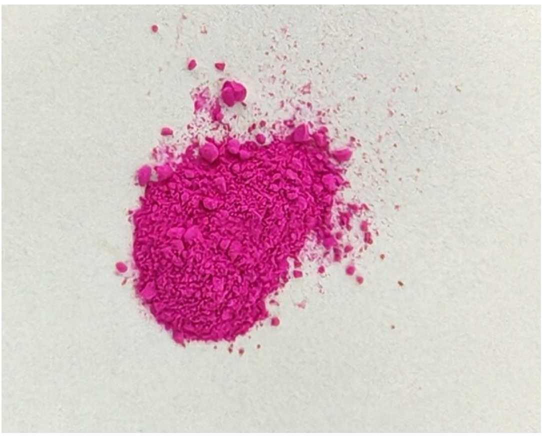

Fig. S5 The color of the mixture of ZIF-8+RB.

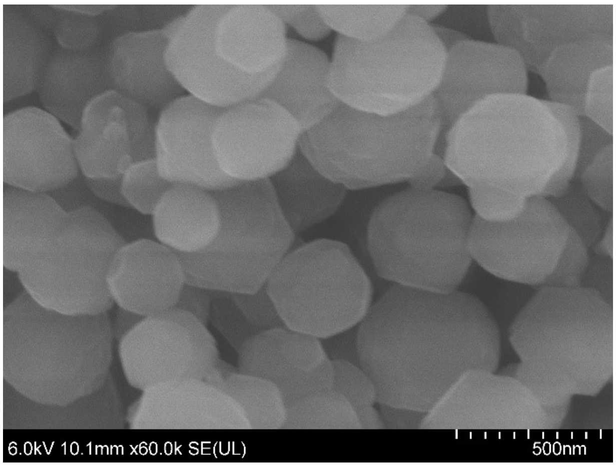

Fig. S6 The SEM of RB@ZIF-8 composite.

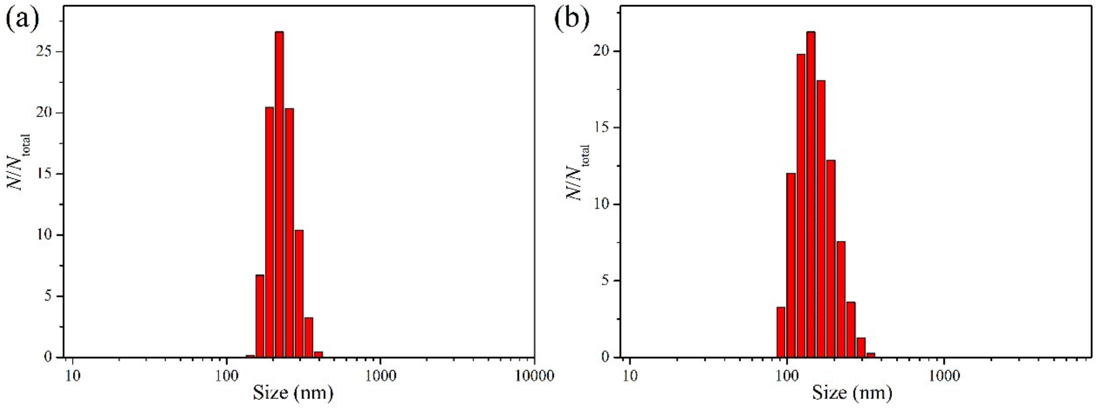

Fig. S7 The size distributions of (a) ZIF-8 and (b) RB@ZIF-8 composite.

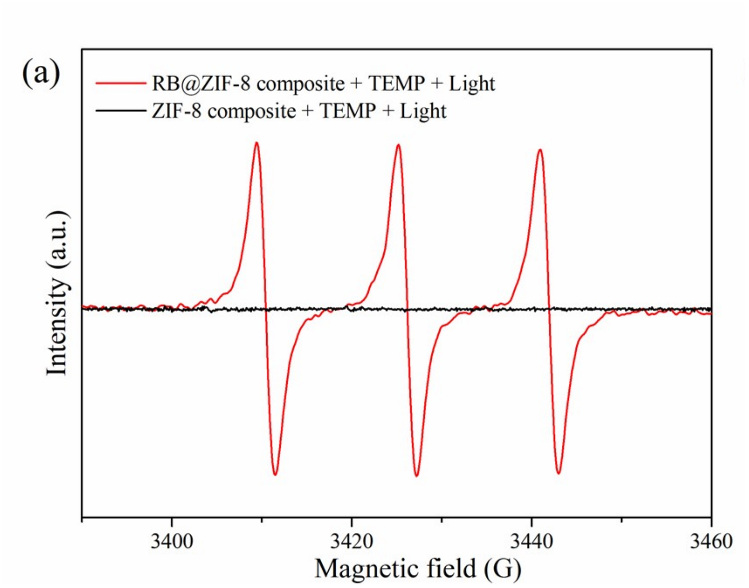

Fig. S8 EPR signals of the TEMP-$^1O_2$ under different conditions.

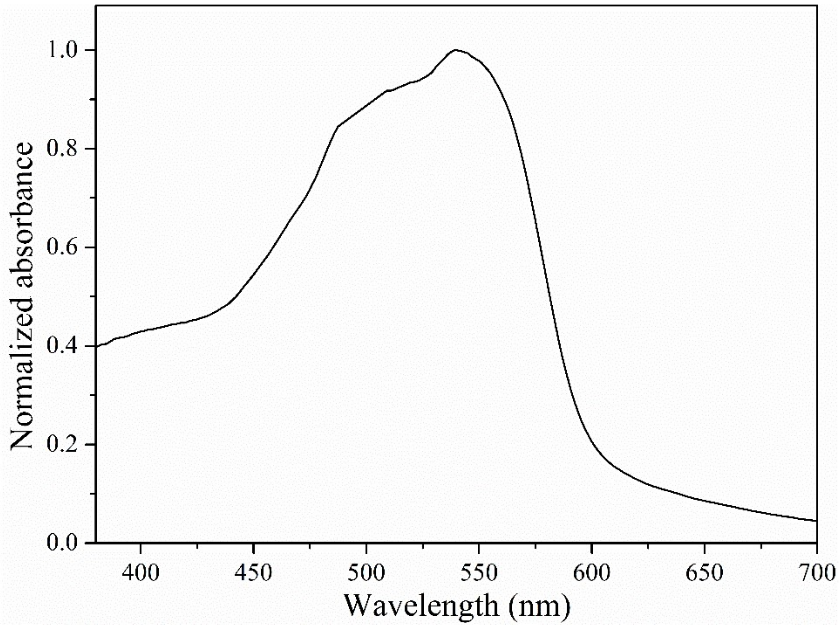

Fig. S9 Solid-state UV-Vis diffused reflectance spectrum of the RB@ZIF-8 composite after catalysis.

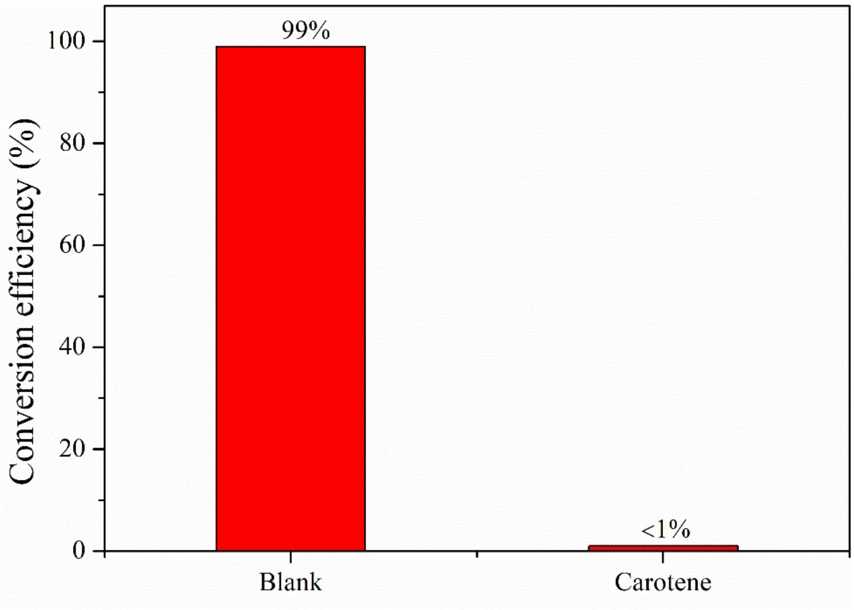

Fig. S10 Conversion efficiency for the detoxification of CEES in the absence or presence of $^1O_2$ scavenger (carotene) upon light irradiation (carotene: 1.0 mmol; RB@ZIF-8 composite: 20.0 mg; CEES: 20 μL; methanol: 1.5 mL; reaction time: 10 min).

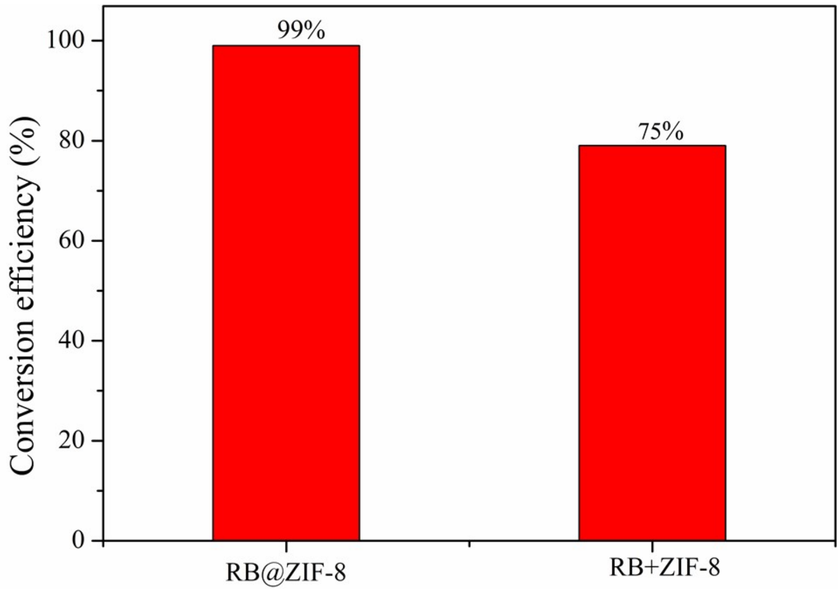

Fig. S11 Conversion efficiency for the detoxification of CEES in the presence of RB@ZIF-8 and RB+ZIF-8 upon light irradiation (RB@ZIF-8 composite: 20.0 mg; RB+ZIF-8 composite: 20.0 mg; CEES: 20 μL; methanol: 1.5 mL; reaction time: 6 min).

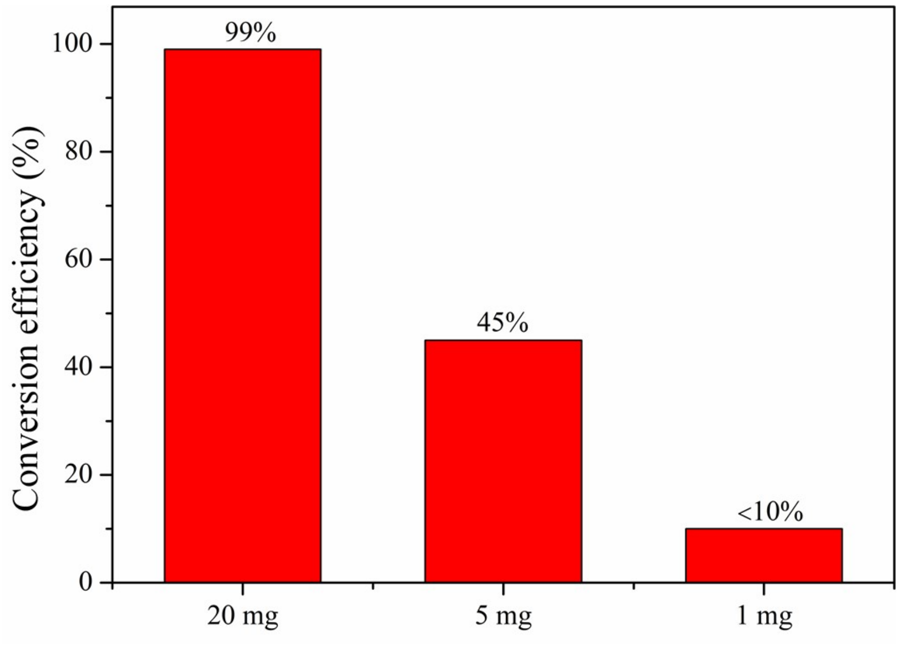

Fig. S12 Conversion efficiency for the detoxification of CEES in the presence of RB@ZIF-8 upon light irradiation (RB@ZIF-8 composite: 20.0 mg, 5 mg and 1 mg; CEES: 20 μL; methanol: 1.5 mL; reaction time: 6 min).

Scheme S1. Proposed mechanism for the photochemical detoxification of CEES by the RB@ZIF-8 composite.

Table S1 Comparison of the performances in the detoxification of of CEES using various MOFs-based heterogeneous photosensitizers.

Catalyst[a]	Solvent	Effective Photosensitizer	Atmo.[b]	Light	Half-life (min)	Ref.

TCPP@MIL-101(Cr)	MeOH	Porphyrin	O_2	Blue LED	1	1
Au/TCPP@MIL-101(Cr)	MeOH	Porphyrin	O_2	Blue LED	0.75	1
Ag_{12}TPyP	CD_3OD	Porphyrin	O_2	White LED	1.5	2
Br-BDP@NU-1000	MeOH	BODIPY	O_2	Green LED	2	3
Br-BDP@NU-1000	MeOH	BODIPY	O_2	Green LED	2.5	3
UiO-68-TBTD	MeOH	TBTD	air	Blue LED	3	4
PCN-67-Se	MeOH	Benzoselenadiazole	O_2	Purple LED	3.5	5
NU-1000-PCBA	MeOH	Pyrene and C_{60}	O_2	UV LED	3.5	6
UMCM-313	MeOH	Pyrene	O_2	Blue LED	4	7
Al-PMOF on fiber	MeOH	Porphyrin	O_2	Blue LED	4	8
I_2-BODIPY@ZIF-8	MeOH	BODIPY	O_2	Green LED	4.5	9
MOF/BA/textile	No solvent	Porphyrin	O_2	Blue LED	4.4	10
MOF/BA/textile	No solvent	Porphyrin	air	Simulated sunlight	17.6	10
Ag_{12}TPyP	CD_3OD	Porphyrin	air	White LED	6	11
NU-1000	MeOH	Pyrene	O_2	UV LED	6	12
PCN-222/MOF-545	MeOH	Porphyrin	O_2	Blue LED	11	12
PCN-57-S	MeOH	Benzothiadiazole	O_2	UV LED	7.5	13
PCN-222/MOF	MeOH	Porphyrin	O_2	Blue (325)	13	14
PCN-222/MOF	MeOH	Porphyrin	O_2	White LED	26	14
PCN-222/MOF	MeOH	Porphyrin	O_2	Red LED	33	14
ZnTTP@ZIF-8	MeOH	Porphyrin	air	Blue LED	1.5	15
MB@UiO-66-$(COOH)_2$	MeOH	Methylene blue	air	Red LED	1.8	16
RB@ZIF-8	MeOH	Rose Bengal	air	Green LED	2.5	This work

[a]The catalysts from literature have been named as published; [b]Atmo.: atmosphere

References:

1 M.-M. Wu, J. Su, D. Luo, B.-C. Cai, Z.-L. Zheng, D.-S. Bin, Y. Y. Li, X.-P. Zhou, Ultrafast photocatalytic detoxification of mustard gas simulants by a mesoporous metal–organic framework with dangling porphyrin molecules, *Small*, 2023, **19**, 2301050.

2 M. Cao, R. Pang, Q.-Y. Wan, Z. Han, Z.-Y. Wang, X.-Y. Dong, S.-F. Li, S.-Q. Zang, T. C. W. Mark, Porphyrinic silver cluster assembled material for simultaneous capture and photocatalysis of mustard-gas simulant, *J. Am. Chem. Soc.*, 2019, **141**, 14505−14509.

3 A. Atilgan, T. Islamoglu, A.J. Howarth, J. T. Hupp, O. K. Farha, Detoxification of a sulfur mustard simulant using a BODIPY-functionalized zirconium-based metal-organic framework, *ACS Appl. Mater. Interfaces*, 2017, **9**, 24555−24560.

4 W.-Q. Zhang, K. Cheng, H. Zhang, Q.-Y. Li, Z. Ma, Z. Wang, J. Sheng, Y. Li, X. Zhao, X.-J. Wang, Highly efficient and selective photooxidation of sulfur mustard simulant by a triazolobenzothiadiazole-moiety-functionalized metal-organic framework in air, *Inorg. Chem.*, 2018, **57**, 4230−4233.

5 S. Goswami, C. E. Miller, J. L. Logsdon, C. T. Buru, Y.-L. Wu, D. N. Bowman, T. Islamoglu, A. M. Asiri, C. J. Cramer, M. R. Wasielewski, J. T. Hupp, O. K. Farha, Atomistic approach toward selective photocatalytic oxidation of a mustard-gas simulant: a case study with heavy-chalcogen-containing PCN-57 analogues, *ACS Appl. Mater. Interfaces*, 2017, **9**, 19535−19540.

6 A. J. Howarth, C. T. Buru, Y. Liu, A. M. Ploskonka, K. J. Hartlieb, M. McEntee, J. J. Mahle, J. H. Buchanan, E. M. Durke, S. S. Al-Juaid, J. F. Stoddart, J. B. DeCoste, J. T. Hupp, O. K. Farha, Postsynthetic incorporation of a singlet oxygen photosensitizer in a metal-organic framework for fast and selective oxidative detoxification of sulfur mustard, *Chem.-Eur. J.*, 2017, **23**, 214−218.

7 C. T. Buru, M. B. Majewski, A. J. Howarth, R. H. Lavroff, C.-W. Kung, A. W. Peters, S. Goswami, O. K. Farha, Improving the efficiency of mustard gas simulant detoxification by tuning the singlet oxygen quantum yield in metal-organic frameworks and their corresponding thin films, *ACS Appl. Mater. Interfaces*, 2018, **10**, 23802−23806.

8 D. T. Lee, J. D. Jamir, G. W. Peterson, G. N. Parsons, Protective fabrics: metal-organic framework textiles for rapid photocatalytic sulfur mustard simulant detoxification, *Matter*, 2019, **2**, 404−405.

9 J.-F. Zhou, J.-J. Ling, G. Li, S. Zhang, D. Zhu, The molecule-level photoreactor:

accurate embedded iodine-substituted boron dipyrromethene dye within zeolitic imidazolate framework-8 for highly efficient oxidization of sulfides under visible light, *Mater. Today Chem.*, 2022, **24**, 100774.

10 Y.-J. Hao, Y. Hao, E. K. Papazyan, Y. Ba, Y.-Y. Liu, Mechanism-guided design of metal organic framework composites for selective photooxidation of a mustard gas simulant under solvent free conditions, *ACS Catal.*, 2021, **12**, 363−371.

11 M. Cao, R. Pang, Q.-Y. Wan, Z. Han, Z.-Y. Wang, X.-Y. Dong, S.-F. Li, S.-Q. Zang, T. C. W. Mark, Porphyrinic silver cluster assembled material for simultaneous capture and photocatalysis of mustard-gas simulant, *J. Am. Chem. Soc.*, 2019, **141**, 14505−14509.

12 C. T. Buru, M. B. Majewski, A. J. Howarth, R. H. Lavroff, C.-W. Kung, A. W. Peters, S. Goswami, O. K. Farha, Improving the efficiency of mustard gas simulant detoxification by tuning the singlet oxygen quantum yield in metal-organic frameworks and their corresponding thin films, *ACS Appl. Mater. Interfaces*, 2018, **10**, 23802−23806.

13 S. Goswami, C. E. Miller, J. L. Logsdon, C. T. Buru, Y.-L. Wu, D. N. Bowman, T. Islamoglu, A. M. Asiri, C. J. Cramer, M. R. Wasielewski, J. T. Hupp, O. K. Farha, Atomistic approach toward selective photocatalytic oxidation of a mustard-gas simulant: a case study with heavy-chalcogen-containing PCN-57 analogues, *ACS Appl. Mater. Interfaces*, 2017, **9**, 19535−19540.

14 Y. Liu, A. J. Howarth, J. T. Hupp, O.K. Farha, Selective photooxidation of a mustard-gas simulant catalyzed by a porphyrinic metal-organic framework, *Angew. Chem. Int. Ed.*, 2015, **54**, 9001−9005.

15 J. Zhou, X. Li, C. Chu, J. Cao, One-pot synthesis of a porphyrin functionalized metal-organic frameworks as a recyclable visible-light-driven photosensitizer for efficient detoxification of a sulfur mustard simulant in air, *Microporous Mesoporous Mater.*, 2024, **375**, 113163.

16 J. Zhou, Q. Zhou, H. Sun, X. Li, A. Chen, J. Chen, C. Chun, Selective detoxification of a sulfur mustard simulant in air by a methylene blue-functionalized metal-organic framework, *Dalton Trans.*, 2025, **54**, 1827–1837.
